# Optimal approach to performing and reporting computed tomography angiography for suspected acute pulmonary embolism: a clinical consensus statement of the ESC Working Group on Pulmonary Circulation & Right Ventricular Function, the Fleischner Society, the Association for Acute CardioVascular Care (ACVC) and the European Association of Cardiovascular Imaging (EACVI) of the ESC, endorsed by European Respiratory Society (ERS), Asian Society of Thoracic Radiology (ASTR), European Society of Thoracic Imaging (ESTI), and Society of Thoracic Radiology (STR)

**DOI:** 10.1093/ehjci/jeaf050

**Published:** 2025-06-03

**Authors:** F A Klok, S Barco, L Bertoletti, S Bhalla, S Dubois, G Le Gal, L B Haramati, E van Hooidonk, M Humbert, S V Konstantinides, I M Lang, W S Murphy, Y Ohno, S Price, M Prokop, P Pruszczyk, A Rossi, P A Thistlethwaite, C West, M Remy-Jardin

**Affiliations:** Department of Medicine—Thrombosis and Hemostasis, Leiden University Medical Center, LUMC Room C-7-68, ALbinusdreef 2, 2300RC Leiden, The Netherlands; Center for Thrombosis and Hemostasis, University Medical Center of the Johannes Gutenberg University, Mainz, Germany; Center for Thrombosis and Hemostasis, University Medical Center of the Johannes Gutenberg University, Mainz, Germany; Department of Angiology, University Hospital Zurich, Zurich, Switzerland; Département of Médecine Vasculaire et Thérapeutique, CHU Saint-Étienne, Mines Saint-Etienne, INSERM, SAINBIOSE U1059, CIC 1408, Université Jean Monnet Saint-Étienne, Saint-Étienne 42055, France; Department of Radiology, Mallinckrodt Institute of Radiology, Washington University, St. Louis, MO, USA; Department of Medicine, Ottawa Hospital Research Institute, University of Ottawa, Ottawa, Ontario, Canada; Department of Radiology and Biomedical Imaging, Yale School of Medicine, New Haven, CT, USA; Université Paris-Saclay, Faculté de Médecine, Inserm UMR_S 999, Service de Pneumologie, Hôpital Bicêtre, Assistance Publique Hôpitaux de Paris, Le Kremlin-Bicêtre, France; Center for Thrombosis and Hemostasis, University Medical Center of the Johannes Gutenberg University, Mainz, Germany; Department of Internal Medicine II, Division of Cardiology, Medical University of Vienna, Center for Cardiovascular Medicine, Währinger Gürtel 18-20, Vienna 1090, Austria; Department of Diagnostic Radiology, Fujita Health University School of Medicine, Toyoake, Japan; Faculty of Medicine, National Heart and Lung Institute, Imperial College, London, UK; Royal Brompton Hospital, National Heart & Lung Institute, Imperial College London, London, UK; Department of Radiology and Nuclear Medicine, Radboud University Medical Center, Nijmegen, The Netherlands; Department of Internal Medicine and Cardiology, Medical University of Warsaw, Warsaw, Poland; Department of Nuclear Medicine, University Hospital Zurich, Zurich, Switzerland; Department of Surgery, University of California, San Diego, La Jolla, CA, USA; Radboud University Medical Center, Nijmegen, The Netherlands; IMALLIANCE Hauts-de-France, Valenciennes, France

**Keywords:** pulmonary embolism, computed tomography, reference standards, outcome, prognosis, diagnosis

## Abstract

Computed tomography angiography (CTA) is the modality used most frequently for diagnosing acute pulmonary embolism (PE). Given the vast amount of information that can be extracted from CTA, the CTA report should be written in a way that conveys all relevant findings using standardized nomenclature and definitions. Broad consensus on a core set of CTA findings that are relevant for all PE patients is not currently available. This clinical consensus statement written by the European Society of Cardiology (ESC) Working Group on Pulmonary Circulation and Right Ventricular Function, the Fleischner Society and the Association for Acute Cardiovascular Care and the European Association of Cardiovascular Imaging of the ESC provides a current update of CTA techniques, a definition of often used nomenclature and recommendations on the proposed content of CTA reports along with a detailed image atlas with instructions on how to assess all relevant CTA findings and a lay language guidance on the meaning of these findings. Ultimately, upon implementation, this document is expected to standardize CTA radiology reports with respect to diagnostic and prognostic CT imaging findings to guide and harmonize management decisions, ultimately improving outcomes of care for PE patients.

## Graphical Abstract

**Graphical Abstract:**
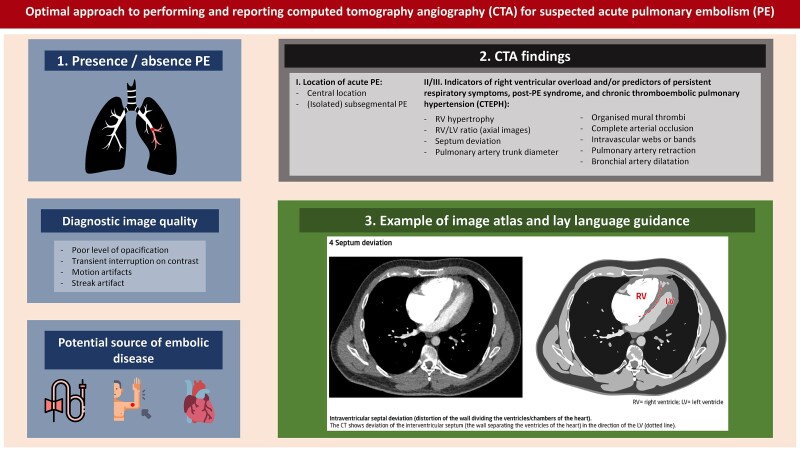

## Introduction

Early recognition and accurate diagnosis of acute pulmonary embolism (PE) is key in the timely initiation of appropriate treatment.^1–5^ Computed tomography angiography (CTA), often referred to as computed tomography pulmonary angiography, is the imaging modality of choice for diagnosing PE if it cannot be ruled out by using a clinical decision-making tool and D-dimer test.^1,2^ In addition to aiding the clinician in the diagnosis of PE, CTA images provide crucial information to guide the initial treatment approach (e.g. parameters of right ventricular overload or dysfunction are well-recognized predictors of short-term prognosis). Moreover, clot morphology as determined by CT imaging has been shown to predict long-term complications of acute PE^1,2,6^ and may guide planning of advanced reperfusion strategies in selected patients. Finally, CTA may suggest alternative diagnoses in patients with dyspnoea or chest pain, such as the finding of pulmonary oedema, pericarditis, or aortic dissection, as well as clues to the underlying cause of the PE (e.g. cancer). Given the broad range of information that can be extracted from a CTA, the report should be timely and structured in a way that conveys all relevant clinical, functional, and prognostically relevant findings.^7,8^ However, to date, a formal standard for reporting a CTA in suspected acute PE derived through expert consensus and distillation of existing evidence does not exist.

For this purpose, representatives of the ESC; Working Group on Pulmonary Circulation & Right Ventricular Function, The Association for Acute Cardiovascular Care, and The European Association of Cardiovascular Imaging of the ESC together with representatives of the Fleischer Society convened to address this unmet need. A multidisciplinary task force was installed with experts in PE diagnosis and management, radiological imaging as well as acute cardiovascular care, all representatives of the above-mentioned scientific societies (50% ESC representation, 50% Fleischer Society representation). The main aims were to produce a consensus statement that (i) describes current CT techniques for diagnosing PE, (ii) provides state-of-the-art best practices to achieve CTA scans with optimal quality, thereby reducing the number of non-diagnostic scans, (iii) standardizes commonly used nomenclature in CTA reports, and (iv) establishes a core set of CTA findings along with assessment instructions that are relevant for all acute PE patients and are advised to be routinely reported.

### Methodology

Initially, the task force undertook an extensive overview of the literature concerning currently available and recommended CT techniques, diagnostic criteria for acute PE, criteria for optimising image quality, and strategies to overcome challenges in achieving diagnostic image quality. A glossary was developed with frequently used terminology to describe the spectrum of pulmonary artery filling defects that can be seen on CTA. The glossary of definitions was extracted from the literature or when not available, determined by task force consensus.

Second, the task force undertook a comprehensive literature search to identify all radiological findings that have been studied for their prognostic relevance for patients with a CTA diagnosis of PE. The search was performed using a predefined search string (see Supplementary data online, *Appendix A*) by 2 independent readers and was integrated by cross-referencing, and additionally included a search of relevant abstracts presented at international congresses. Relevant papers were screened, identified, and summarized in predefined outcome tables per radiological parameter. Selection of the core set of radiological findings relevant for all PE patients was undertaken in an online three-round modified Delphi process: all CTA findings that were identified in the literature search to be consistently demonstrated to have prognostic value for the short (first 3 months after diagnosis) and/or long-term (beyond the first 3 months of follow-up) prognosis of PE patients were eligible. The following adverse events were deemed to be relevant for PE prognosis: death, admittance to the intensive care unit, haemodynamic collapse, right ventricular failure, respiratory insufficiency, need for cardiopulmonary support, bleeding, recurrent venous thromboembolism, symptom burden, persistent symptoms, chronic thromboembolic pulmonary disease with/without pulmonary hypertension, poor quality of life, change in New York Heart Association functional class, post-PE syndrome, post-PE impairment, myocardial infarction, stroke, systemic embolism, and health care costs. Using the RAND–University of California (Los Angeles, CA) method to reach consensus, all task force members were required to vote (excluding the patient representatives) to determine which outcomes should be kept for assessment in relation to the radiological findings.^9^ The results of each vote were reviewed by all voting members of the task force before the next voting round. Inclusion in the final core set required that at least 80% of the task force voted an item as essential. Essential CTA findings were subdivided into ‘must-haves’ and ‘nice-to-haves’. The latter indicated that the finding was considered relevant but not critical. CTA findings were excluded if at least an 80% majority voted an item as not advised. In the last voting round, a 70% majority was accepted as decisive. Lastly, an image atlas was created to illustrate the identified core set of CTA findings, including instructions on how these are to be assessed.

From inception, four patient representatives were involved in the planning of the project and task force activities, not only providing the patient perspective but also providing text for the lay language version of the imaging atlas.

### CT scanning technology

#### Chest CTA with single-energy CT

Over the last decades, the role of CT in the diagnostic approach of acute PE has exclusively relied on single-energy CT. The continuous technical evolution of multi-detector row CT (MDCT) has allowed for more flexible and high-performance protocols that match diagnostic needs without compromising detail and coverage. The precise scan parameters depend on the number of slices that can simultaneously be acquired, gantry rotation speed, table speed, and beam width. Protocols differ according to the number of X-ray sources, the number of detector rows (currently ranging from 16 to 320 detector rows). The radiation dose of chest CTA examination has been reduced by the introduction of low-kilovoltage (kV) scanning. Hybrid- or model-based iterative reconstructions or deep learning reconstructions have been shown to reduce noise better than the more traditional filtered back projection and are considered the current technical standard.^10,11^

Whatever the CT equipment available, the choice of scanning parameters follows universal recommendations. First, the highest temporal resolution (i.e. shortest rotation time) should be selected to avoid respiratory motion artefacts and minimize cardiac motion artefacts. Second, the entire thorax should be covered at the highest pitch for non-pregnant persons, keeping in mind that the entire thorax can be currently covered in less than one second with high-pitch scanning modes. This can be of major interest in the emergency department, intensive care, or for elderly patients where apnoea cannot be sustained. Shallow breathing is preferred to breath-hold techniques in dyspnoeic patients for better tolerance. Low-kV imaging is advised as it increases intravenous contrast material attenuation and minimizes radiation dose.^12^ The optimal selection can be obtained using weight-based tube voltage selection or by means of automatic tube voltage selection.^13^

Examinations are typically obtained using automatic triggering of data acquisition when the attenuation within the vessel of interest reaches a pre-defined threshold (i.e. timing bolus or bolus tracking). The traditional CTA examination relies on the positioning of a region of interest (ROI) at the level of the pulmonary trunk with an exclusive opacification of the pulmonary arterial circulation at an optimal phase. Exclusive opacification of the pulmonary arterial circulation is not encouraged because (i) the absence of opacification of the systemic circulation (aorta) does not give access to aortic differential diagnoses in the event of chest pain, (ii) in case of retrograde systemic- to-pulmonary artery shunts, very misleading images simulating filling defects may be observed, and (iii) hypertrophy of the bronchial arteries (visible only in cases of global pulmonary and systemic opacification) can help detect chronic thromboembolism pulmonary hypertension (CTEPH).

An additional option is to scan the entire thorax while the pulmonary and systemic arterial circulations are simultaneously opacified, both at an optimal phase; the ROI is positioned at the level of the ascending aorta or left atrium. This technique simultaneously allows for detection of endovascular pulmonary clots, and a comprehensive analysis of the chest organs at a systemic arterial phase with a single acquisition. It also provides adequate enhancement of cardiac cavities that may harbour acute thromboembolic disease. Contrast enhancement of the vessels is mainly determined by the iodine concentration and injection rate (iodine flux). A rapid uniphase injection bolus is needed to achieve a high intensity of contrast opacification.^14^  *Table 1* summarizes the key scanning parameters for an optimized CTA examination in adults. All examinations are standard, non-electrocardiogram (ECG)-gated acquisitions. Of note, a recent expert consensus document of the Society of Cardiovascular Computed Tomography suggests that it would be appropriate to ECG gate aortic dissection, aneurysm and PE CTA examinations in men > 45 years and women >55 years to analyse and report the coronary arteries, a recommendation that was followed by the European Association of Cardiovascular Imaging (EACVI).^15,16^

**Table 1:** Optimal CTA scan protocol for routine clinical practice

**Acquisition parameters**	
Tube voltage	80–140 kV according to BMI Possibility to use automatic kV selection (*low kV in slim patients*)
Tube current	Tube current varies based on the kilovoltage, patient size and region thickness (*image quality factor determined in protocol*) Use of automatic tube current modulation
Rotation time	Shortest rotation time
Collimation	Thinnest collimation
Scanning direction	Cranio-caudal or caudo-cranial Alternative for severely dyspnoeic patients: caudo-cranial imaging to decrease respiratory motion artefacts in the lung bases
Respiratory instructions	Breath-hold at deep inspiration Breath-hold at vital capacity or shallow breathing if chest pain/dyspnoea
**Reconstruction parameters**	
Section thickness	1-mm thick transverse CT sections
Field of view	Adapted to the patient size
Kernels of reconstruction	Soft-tissue and high-spatial-resolution kernels for mediastinal and lung images, respectively
Reconstructions	Iterative reconstruction or deep learning reconstruction
**Injection parameters**	
Venous access	> 20G in antecubital vein; smaller if poor venous access
Flow rate	3–5 mL/s in antecubital vein; lower if poor venous access *(with higher iodine concentration)*
Concentration of the iodinated contrast material	300–370 mg I/mL
Volume administered	80–100 mL

In pregnant patients, larger blood volumes and increased cardiac output can lead to earlier contrast opacification of the pulmonary arteries and dilution of contrast.^17,18^ Poorly enhanced normal vessels can be misinterpreted as PE or fail to allow for visualization of PE. Tailoring CTA protocols in this patient population is essential to optimize contrast opacification and minimize radiation exposure to both the patient and foetus. Scanning the patient from the diaphragm to the top of the aorta reduces radiation exposure by 70%, without sacrificing important diagnostic information.^19^  *Table 2* highlights CTA protocol optimization for PE in pregnant patients. When applied in a prospective trial, the number of non-diagnostic scans was very low.^20^

**Table 2:** Special considerations in pregnant patients

Pregnancy-related concerns	Protocol	
Radiation dose reduction to maternal breast tissue and fetus	Tube voltage	Use of low (80–100) kV
	Tube current	100 kV and fixed mAs value around 80–100 mAs for a non-obese women
Constant factors	Field of view	Adapted to the patient size
	Rotation time	Shortest rotation time
	Breathing	Breath-holding after mild inspiration (vital capacity) or alternatively simple apnoea with an open mouth to avoid Valsalva
	Scanning direction	Caudocranial imaging to decrease respiratory motion and dense contrast in SVC
	Section thickness	1-mm section thickness
	Reconstruction kernels	Soft tissue (mediastinal images) and high-spatial frequency (lung images) kernels
Poor opacification based on expanded blood volume, hyperdynamic state and higher risk of transient interruption of contrast	Contrast media injection protocol	> 20G intravenous access in antecubital vein Higher flow: 5 mL/s Higher contrast concentration than standard protocol Patient coaching to minimize contrast interruption

Adequate contrast opacification is critical for diagnostic quality, which depends upon patient weight, cardiac output, scan duration, breath holding, and contrast delivery protocol.^14,17,18,21–29^ The objective is to obtain homogenous opacification of pulmonary arteries that optimizes the diagnostic image quality for detection of acute PE. Arterial enhancement depends on the amount of contrast delivered per unit of time (injection flow rate) and the injection duration, measured in seconds. With early MDCT scanners (i.e.16-MDCT), the theoretic minimum attenuation of blood required to see acute thromboemboli up to the segmental level was calculated to be 93 HU, a threshold not applied in clinical routine.^30^ In current practice, on a 64-detector CT, a mean pulmonary artery opacification of 250 HU can be achieved with 1.2 mL/kg of 350 mg I/mL injected at 4 mL/s.^14^ The scan duration depends upon the scanner. With a faster scanner, contrast volume can be decreased but timing becomes more crucial as the bolus duration shortens.^31^

Although a contrast flow rate of at least 3 mL/s is associated with a lower frequency of insufficient contrast enhancement during chest CT, a flow rate of more than 4 mL/s using an 20-gage (G) cannula has been suggested optimal for pulmonary thromboembolism (5, 21).^14,32,33^ A lower volume of contrast and iodine dose can be administered when using a higher iodine concentration (350 mg iodine/mL vs. 300 mg/mL) without compromising diagnostic image quality.^34,35^ Intravenous contrast needs to be adjusted for patients with higher body mass index (BMI) (starting with a BMI exceeding 30 kg/m^2^).^26^ Increasing contrast flow rate to ideally around 5–6 mL/s, increasing contrast volume to at least 90 mL and using higher contrast concentration are effective in improving pulmonary arterial enhancement.^36^ To minimize the risk of contrast extravasation, unnecessary high injection pressure and increased contrast viscosity, an 18-G peripheral venous catheter or, even better, a 20-G fenestrated peripheral venous catheter is placed in an antecubital position. Fenestrated catheters are more recently available peripheral intravenous access devices with multiple side holes as opposed to a single end hole of standard catheters. While their smaller size improves the successful placement rate especially in patients with smaller veins, the 20-G fenestrated catheter allows an infusion rate as high as a traditional 18-G catheter at 5.0–7.5 mL/s.^37^

#### Chest CT angiography with spectral imaging and subtraction imaging

Dual-energy CT (DECT) involves the acquisition of two or more CT measurements with distinct energy spectra. Using the differential attenuation of tissues and materials at different x-ray energies, DECT allows distinction of tissues and materials beyond that possible with conventional CT.^38^ DECT technologies can operate at the source or detector level. Dual-source, rapid tube-voltage switching, and dual-layer detector CT are the most commonly used DECT technologies. Although contrast-to-noise levels for unfiltered DECT data vary, increasing with increasing energy separation, final image quality is mainly determined by the sophisticated noise suppression algorithms used by all vendors. Most of the currently available technologies typically use two energy levels, commonly referred to as DECT. Radiation dose with DECT is similar to that with single energy CTA acquired at 120 kV but usually higher than that of CTA acquired at lower kV.

With the use of two or more energy bins, photon-counting detector CT (PCD-CT) can provide the same information as multi-energy CT. Preliminary studies suggest that PCD-CT can perform routine CT with similar image quality and lower radiation dose as compared with DECT.^39–46^ Optimized protocols for chest CT angiography with PCD-CT combine ultra-high-resolution and high-pitch data acquisition that improve the overall image quality at lower radiation doses compared with traditional scanners equipped with energy-integrating detectors.^47,48^ As these approaches allow the creation of perfused blood volume images, a higher concentration of iodine (350–400 mg/mL) is usually advised for the injection protocol.

Subtraction imaging also relies on the acquisition of two measurements: one before contrast injection and one at peak contrast enhancement. A low-kV technique is used and the pre-contrast scan is performed with a 30–50% lower dose than the post-contrast scan. By subtracting the pre-contrast from the post-contrast scan, an iodine enhancement map is created requiring a non-rigid registration to accommodate changes in inspiration. In addition, vessels are masked out and image noise is suppressed to provide a parenchymal enhancement map, similar to ones employed with dual-source DECT.^49–51^ Analogous to DECT, scans have to be timed so that there is already substantial pulmonary venous enhancement in addition to pulmonary arterial enhancement, to ensure that the capillary bed of the lung is sufficiently enhanced and perfusion differences can be reliably detected.

CT lung perfusion imaging does not correspond to a comprehensive blood flow analysis but provides a static, single time point map of the iodine content within the distal pulmonary bed (i.e. arterioles, capillaries, and venules) after injection of contrast material. It differs from true perfusion imaging, which requires imaging of an anatomic ROI at a series of time points or from nuclear perfusion imaging which relies solely on pulmonary arterial supply.^52^ CT lung perfusion with the CTA protocol is a snapshot of iodine distribution within distal pulmonary arteries and capillaries. CT lung perfusion with a general CTA protocol provides information at a later time point, thus showing systemic-to-pulmonary artery shunts in case of abnormal systemic collateral supply. The images can be generated as grey-scaled or as colour-coded images. The perfusion maps can be fused with conventional CT images, allowing simultaneous assessment of morphological and physiologic information.^53,54^ In the context of acute PE, CT lung perfusion imaging improves the diagnostic capabilities of CT by demonstrating PE-type defects (i.e. pleural-based, triangular defects) as well as obstructive clots, enhancing the detection of peripheral clots.^55^

Based on the technique of two-material decomposition into a soft tissue image and an iodine image, virtual monoenergetic images (VMI) are generated which demonstrate how the image object, in particular the iodine, would look if the X-ray source produced X-rays at a single energy level. In the context of acute PE, VMIs at low energy (usually 45–55 keV) result in increased iodine attenuation, and thus detection of endoluminal filling defects. An additional advantage of monochromatic imaging is the reduction of iodine load for routine chest CT angiographic examinations. Reduction of iodine load may be critically important for reducing post-imaging renal impairment in patients with moderate or severe renal insufficiency.^56,57^

#### CT venography

Lower extremity ultrasound is the preferred method of evaluation for deep vein thrombosis (DVT). It is radiation-free, readily available, and inexpensive.^58^ Indirect CT venography (CTV) of the abdomen, pelvis, and lower limbs to search for concomitant DVT is possible after CTA but is rarely performed in the setting of (suspected) acute PE. For conventional CTV non-helical sequential scanning slice widths between 5 and 10 mm and slice intervals up to 5 cm are used. This technique bears the risk of missing shorter segmental clots.^59^ Modern helical technique uses 2.5–10 mm reconstructed image thickness with maximum table speed possible and carefully adjusted mA settings with automated dose modulation protocols. Scanning should include the deep venous system from the inferior vena cava (IVC) and iliac veins down to the popliteal fossae. Ideal scan delay is usually 180 s after the beginning of a larger contrast bolus administration than CTA. Shorter delays can lead to false-positive pseudo-thrombotic findings due to incomplete recirculation and uneven mixing of injected contrast into the venous blood pool.^60–63^ Elastic stockings have been recommended to enhance deep venous filling, but this is not a practical option, particularly in critically ill patients.^63^ CTV should not be part of routine care, but may be considered in rare situations where, for instance, CUS is not feasible or available, i.e. rare venous malformations or extreme morbid obesity, or in case of suspected inferior vena cava or iliac vein thrombosis.

#### Diagnostic image quality

The major criteria for diagnostic image quality in this setting are adequate arterial enhancement on images devoid of motion artefacts. A non-diagnostic CTA examination secondary to insufficient image quality should be repeated to secure a final diagnosis regarding the presence or absence of PE, to avoid under- and overtreatment. The scan should be analysed to detect the cause of suboptimal image quality, and the circumstances/scan parameters should be revised accordingly. *Table 4* summarizes the most frequent causes of non-diagnostic CTA scans and provides solutions to improve image quality.

**Table 3:** Relevance of the level of analysability of the pulmonary arteries for the diagnostic value of the CT

Mediastinal images		Perfusion images	Diagnostic value of CT
Level of analysability of pulmonary arteries	Diagnostic value of mediastinal images alone		Based on combined reading of mediastinal and perfusion images
PAs analysable down to the subsegmental level	Negative down to the subsegmental level	No segmental/subsegmental perfusion defects	Truly negative
PAs analysable down to the segmental level	Negative down to the segmental level (*does not exclude subsegmental clots*)	No *segmental/subsegmental* perfusion defects	Truly negative
		Presence of *segmental/subsegmental* perfusion defects	Peripheral acute PE (after combined assessment of perfusion and morphology)
Only central PA analysable	Only central clots excluded	No diagnostic value of perfusion images	Indeterminate for PE
PAs not analysable	PE not excluded	No diagnostic value of perfusion images	Non-diagnostic examination

**Table 4:** Causes of and solutions to nondiagnostic CTA

Finding	Potential aetiology	Proposed solution
Poor level of opacification within PAs	Incorrect placement of the ROI	Repeat after correct positioning of the ROI
	Inadequate HU threshhold to start exam	Repeat after correction
	Delayed transit time Due to low cardiac output (most often RV) Due to increased pulmonary vascular resistance (acute airspace disease; focal lung abnormalities)	Repeat After selecting a longer start delay (if CTA) Repeat with a ROI in the ascending aorta/left atrium (general CTA protocol)
	Dilution of the column of contrast Due to patent foramen ovale Septal defects Retrograde systemic-to-pulmonary artery shunt	Repeat after mild inspiration No attempt to repeat^a^ No attempt to repeat^a^
Transient interruption on contrast (TIC)	Deep inspiration in a patient with large volume of unopacified blood from the inferior vena cava diluting the bolus of contrast material	Repeat using mild inspiration or simple apnoea
	Veno-arterial extracorporeal membrane oxygenation (ECMO) may cause changes in the filling of the pulmonary arteries	ECMO flow should be reduced to a minimum during the CTA
Motion artefacts	Causes: Respiratory motion Patient movement during the examination Cardiac motion: usually most pronounced in left lower lobe and lingula and on older scanners	Repeat (caudo-cranial) if patient cooperation possible Repeat (caudo-cranial) with shallow breathing if patient cooperation is impaired Repeat (caudo-cranial) if patient cooperation possible No attempt to repeat
Streak artefact	Beam-hardening artefacts due to: Dense contrast material Metal implants or bullets	No attempt to repeat Post-processing with algorithms suppressing metal artefacts (*manufacturor dependent*) or reconstruction of high-energy images (if acquisition with DECT/PCD-CT)

^a^ *Potential alternative*: administration of more contrast (120–150 mL), at low flow rate (3 mL/s) with a long start delay (>30 s) to ensure opacification of pulmonary arteries as well as shunt veins.

Adequacy of pulmonary artery opacification in clinical practice is usually performed qualitatively, relying on a homogeneous column of contrast within the arterial lumen. Pulmonary artery opacification can also be quantitatively assessed by measuring the attenuation values within central and peripheral pulmonary arteries. A described minimum threshold is ∼200–250 HU. Simultaneous reading of lung and mediastinal images is mandatory when analysing segmental and subsegmental pulmonary arteries to make sure that breathing or cardiogenic motion artefacts mimicking endoluminal clots are ruled out, although this is not always achievable. Because image graininess due to image noise can alter the diagnostic value of images, one should anticipate this as a potential cause of reduced image quality and carefully select scanning parameters, in particular the kilovoltage. These challenges tend to be more difficult to mitigate in the relatively common scenario of patients with conditions associated with elevated risk of PE, such as severe illness, obesity, and pregnancy.^64^ Whereas the detection of endoluminal clots allows an easy diagnosis of acute PE, the level of analysability of pulmonary arteries is relevant for the diagnostic value of the negative pulmonary CTA examination and should be included in the CTA report (*Table 3*). If only the central pulmonary artery can be evaluated, the scan is ‘indeterminate for PE’. More commonly, in degraded studies some segmental arteries remain analysable, while others are not. Such regional suboptimal enhancement usually has an explanation such as parenchymal compression-infiltration, which should be stated in the report.

Despite technological advances, the CTA can occasionally be nondiagnostic. *Table 4* highlights potential causes for nondiagnostic examinations and corrective measures that we proposed to restore a diagnostic image quality. Competitive inflow of unopacified blood from IVC can limit pulmonary artery opacification due to mixing with opacified blood from the superior vena cava (SVC) during deep inspiration. This can manifest as ‘transient interruption of contrast’, observed after deep inspiration that increases the intrathoracic negative pressure with subsequent inflow of unopacified blood from the IVC into the right heart and pulmonary circulation. Transient interruption of contrast is suggested when dense contrast is visualized in the SVC in the setting of unopacified blood within segments of pulmonary artery and the right heart.^23,30^ Another cause of poor opacification within pulmonary arteries can be observed after deep inspiratory breath hold with a Valsalva manoeuvre. During this manoeuvre, there may be a shunt of contrast material from the right to the left atrium through a patent foramen ovale, recognized by the simultaneous finding of poor pulmonary artery enhancement and dense opacification of the aorta. In both situations, technologists should coach patients to maintain a shallow breath hold at the time of the repeat examination. In patients too dyspnoeic to hold their breath, acquisitions at shallow free breathing are alternatives to evaluate central pulmonary arteries.^24^ Model-based iterative image reconstructions and deep learning reconstructions can substantially reduce image noise without dose increase. Increasing tube current, slowing the gantry rotation time and lowering the table’s pitch can increase the signal-to-noise ratio, especially in situations in which a substantial increase of radiation exposure is required, for example, due to morbid obesity.^26,28^ A lower pitch prolongs the study and can also exacerbate motion artefacts. Automatic tube current modulation technology is available on most scanner manufacturers to assess body size from the scout image and modulate the tube current in the *x*, *y*, and *z* axes to achieve a prescribed image quality. Finally, veno-arterial extracorporeal membrane oxygenation causes changes in the filling of the pulmonary arteries when given at high volumes; therefore, its flows should be temporarily halted or reduced to a minimum during the execution of CTA.

Recently, artificial intelligence has shown promising accuracy for the diagnosis of acute PE. It has also shown promise in detecting acute on chronic PEs and in risk stratification. These have the potential to reduce time to diagnosis, facilitate the activation of PE response teams, and increase the reproducibility of the diagnosis.^65^ A rapid evolution of artificial intelligence algorithms in patients with suspected or confirmed PE has to be expected in the years to come.

### Diagnosis of acute PE on CT

The diagnosis of acute PE rests on the detection of a well-defined area of low attenuation in the enhanced pulmonary artery. The filling defect should be identifiable on more than one plane and on several consecutive slices, and should have distinct borders in order to avoid confusion with flow. Many radiologic signs have been used in diagnosing acute PE, including the rim sign which refers to contrast surrounding a central filling defect and the tram-track or railway sign referring to a vessel imaged in plane where the contrast around a PE takes on the appearance of train tracks.^66^ Images should be windowed at the picture archiving and communication system workstation so that the reference intravascular structures can be seen (e.g. the moderator band). If these structures can be viewed, then a PE should be visible. Most acute PE will be central within the vessel lumen. When eccentric, acute PE tend to form acute angles with the vessel wall. Acute PE may locally distend the vessel, especially when it is occlusive. Occlusive PE will lead to a defect on lung perfusion images from DECT or subtraction CT. A reduction in pulmonary blood volume can be seen if a clot leads to a major obstruction but no occlusion.

Additional findings supportive of a PE diagnosis can be seen on lung windows. Pulmonary infarcts manifest as peripheral airspace opacities that have heterogeneous decreased or absent enhancement compared with atelectatic lung.^67–69^ They may present with an internal ground-glass or sponge-like appearance with a rim of more well-defined consolidation. This manifestation is sometimes referred to as the reversed halo sign and reflects the dual blood supply to the lung with foci of preserved non-infarcted lung adjacent to infarcted lung. On lung perfusion images from DECT or subtraction CT pulmonary infarcts show no enhancement within but sometimes increased enhancement in the immediately adjacent normal lung. Very rarely, an area of lung with decreased attenuation will be seen with the classic radiographic Westermark sign. Most often, no parenchymal abnormality is detected. Of note, mosaic attenuation and enlarged bronchial arteries are not seen in the context of acute PE alone. Their presence should prompt detailed search of vascular features of chronic thromboembolic pulmonary vascular disease (CTEPD) with or without pulmonary hypertension that might suggest acute PE superimposed on CTEPD.^70–78^ Any CT performed for PE should also address any potential source of embolic disease. These include central catheters, upper extremity venous thrombosis or cardiac cavities. *Table 5* highlights the definition of often used terminology in CTA reports.

**Table 5:** Continued developments in CT technology have led to marked improvement in spatial resolution and improved detection of all types of intraluminal filling defects on CT pulmonary angiography. However, not all filling defects represent acute pulmonary emboli. The following glossary of CT findings define the spectrum of pulmonary artery filling defects

Term	Definition
Acute PE	Acute PE is characterized by filling defects within the pulmonary arteries that essentially retain the cylindrical shape of the dislodged thrombi that either may remain intact or may fragment on their way through the heart. On images perpendicular to the vessel, acute emboli present as round filling defects in the centre of a pulmonary artery or, when eccentric, they tend to form acute angles with the vessel wall. No signs of deformation due to (auto)lytic activity of the embolus are seen. Acute emboli may totally occlude a pulmonary artery. No contrast enhancement is seen beyond this point. As a result, the diameter of the artery expands to its maximum, usually larger than arteries of comparable anatomic locations elsewhere. In non-occlusive emboli, flow may be seen distal to the filling defect. These tend to be more common at arterial bifurcations.
Central PE	PE is located in either the pulmonary trunk, main pulmonary artery, or lobar pulmonary artery.
Chronic PE	Persistent filling defects after acute PE. The cross-section of these filling defects is thread-like, band-like, web-like, or irregular but not round. Round cross-sections usually suggest recurrent acute PE.
Embolus	An embolus is a thrombus that has dislodged and is located at a site different from its vessel of origin.
Incidental PE	PE identified on a CT scan ordered for any other reason than suspected PE.
In-situ pulmonary thrombus	In-situ pulmonary thrombus presents as a wall-adherent filling defect, usually in large or enlarged pulmonary arteries. The most common location is along the superior aspect of the main right pulmonary artery and intralobar pulmonary artery. These filling defects may be associated with some calcification. Unlike with chronic pulmonary emboli, mosaic attenuation is unusual. It oftentimes occurs in the setting of slow flow and pulmonary hypertension. They are commonly seen in various causes of pulmonary hypertension, most notably Eisenmenger syndrome in the setting of a longstanding right-to-left shunt.
Isolated subsegmental PE	Subsegmental PE limited to a single subsegmental artery.^79^
Peripheral PE	PE located in a segmental or more distal pulmonary artery.
Pulmonary artery filling defect	Pulmonary arteries on contrast-enhanced CT normally show homogeneous contrast enhancement. Filling defects are areas within an affected pulmonary artery that show no contrast enhancement while other pulmonary arteries do enhance. Pulmonary artery filling defects are caused by pulmonary emboli, pulmonary thrombosis, or intravascular tumour.
Pulmonary thrombosis	Thrombi in the pulmonary arteries are usually the result of venous thromboembolism, but may also be local pulmonary thrombosis. This has been recognized in patients with pulmonary hypertension, chronic obstructive pulmonary disease, sickle cell disease, and quite extensively in patients with COVID-19. Differentiation between venous thromboembolism and *in situ* thrombosis can currently not be reliably made based on CTA.
Reduced pulmonary contrast enhancement	Filling defects must be distinguished from regions of reduced contrast enhancement due to early timing of a scan series on contrast-enhanced CT or due to slow flow. They are artefacts associated with scan timing or patient factors (*breathing or pulsation artefacts*). The measured CT attenuation is higher than that of an unenhanced pulmonary artery but lower than that of a well-enhanced vessel.
Right ventricular dilatation	A number of methods have been studied to define right ventricular dilatation based on CTA findings with or without ECG alignment. A practical approach consists of measuring the largest ventricle diameters in the axial view in both ventricles, as defined by the distance between the endocardium and the interventricular septum. As a confirmation, the same measurement can be done taking the largest ventricle diameters at the level of the heart valves. RV/LV ratio measured at CT may be different from measured at echocardiography when the CT is performed without ECG alignment.
Right ventricular hypertrophy	Increased thickness of the right ventricle free wall, exceeding 4 mm.
Saddle embolism	A large central PE that straddles the pulmonary trunk bifurcation.
Subacute PE	The shape of the emboli begins to change due to lytic activity. Instead of a round cross-section, concave portions max occur, and the cross-section become increasingly irregular. Findings of chronic emboli (namely, mosaic attenuation and enlarged bronchial arteries) are usually absent.
Subsegmental PE	A contrast defect in a subsegmental artery, i.e. the first arterial branch division of any segmental artery independent of artery diameter, visible in at least two consecutive axial slices, using a CT scanner with a desired maximum collimator width of ≤1 mm.^79^
Thrombus	An intravascular blood clot that remains at its site of origin.

### Delphi

A literature review (see Supplementary data online, *Appendix A*) was performed to identify all prospective or retrospective studies reporting on prognostic relevance of any CTA findings; studies in less than 100 patients were excluded. The search was performed on 1 June 2022 and yielded 589 articles. After screening the literature search results and integrating with the results of cross-referencing and personal communications from the members of the task force, 43 studies fulfilled our inclusion criteria for at least one outcome. The correlations of the different CTA findings with either short- or long-term prognosis as described in those papers were summarized (see Supplementary data online, *Appendix B* and *C*) and discussed by the task force.^50,72,80–110^ Duplication of data results between individual studies and meta-analysis was minimized. The studies involved mostly haemodynamically stable patients with low rates of adverse events. CT scan techniques, assessment of the CTA findings, thresholds of measurements as well as clinical outcomes and duration of follow-up were highly variable across the studies. Independent adjudication of outcomes was usually lacking and reproducibility of the assessment of the CTA findings was only rarely reported. More often, the reported multivariable models did not include all possible relevant CTA findings; we could therefore not determine which parameters were truly independent predictors of prognosis. Based on the results from the literature, the task force agreed on the selection of 14 CTA findings for the consensus process: those were consistently reported to have a positive association with the prognosis of PE patients and assumed to be easily assessed manually without the need for dedicated experience (*Table 6*).

**Table 6:** Result of the delphi analysis on core CTA findings to be included in CTA radiology reports: percentage agreement is indicated. In the first 2 rounds, agreement wat defined as an 80% majority, in the third round, this was 70% majority

CTA findings	Excluded	Included	Specifics
1. RV/LV ratio (axial images)		100%	92% ‘must have’
2. Central location		100%	85% ‘must have’
3. (Isolated) subsegmental PE		92%	81% ‘must have’
4. Septum deviation		85%	81% ‘must have’
5. IVC reflux	81%		
6. PA trunk diameter		81%	75% ‘nice to have’
7. Coronary artery calcification score	81%		
8. Qanadli score	85%		
9. Organized mural thrombi		85%	75% ‘nice to have’
10. Complete arterial occlusion		94%	75% ‘nice to have’
11. Intravascular webs or bands		100%	75% ‘must have’
12. Pulmonary artery retraction		87%	75% ‘must have’
13. Bronchial artery dilatation		87%	80% ‘must have’
14. RV hypertrophy		94%	75% ‘must have’

During the consensus process the task force members were reminded before each voting round to be mindful of time (how long it takes to assess the parameter), required expertise (some variables can possibly not be assessed in a valid and reproducible way by less experienced radiologists during busy shifts) and the interobserver agreement as presented in the studies we identified. Ultimately, eight CTA findings were considered ‘must-haves’ and three ‘nice-to-haves’. The remaining three were not considered sufficiently indicative and included the following: inferior vena cava reflux, the Qanadli score to quantify thrombus burden and the coronary artery calcification score.^111^

**Figure 1:**
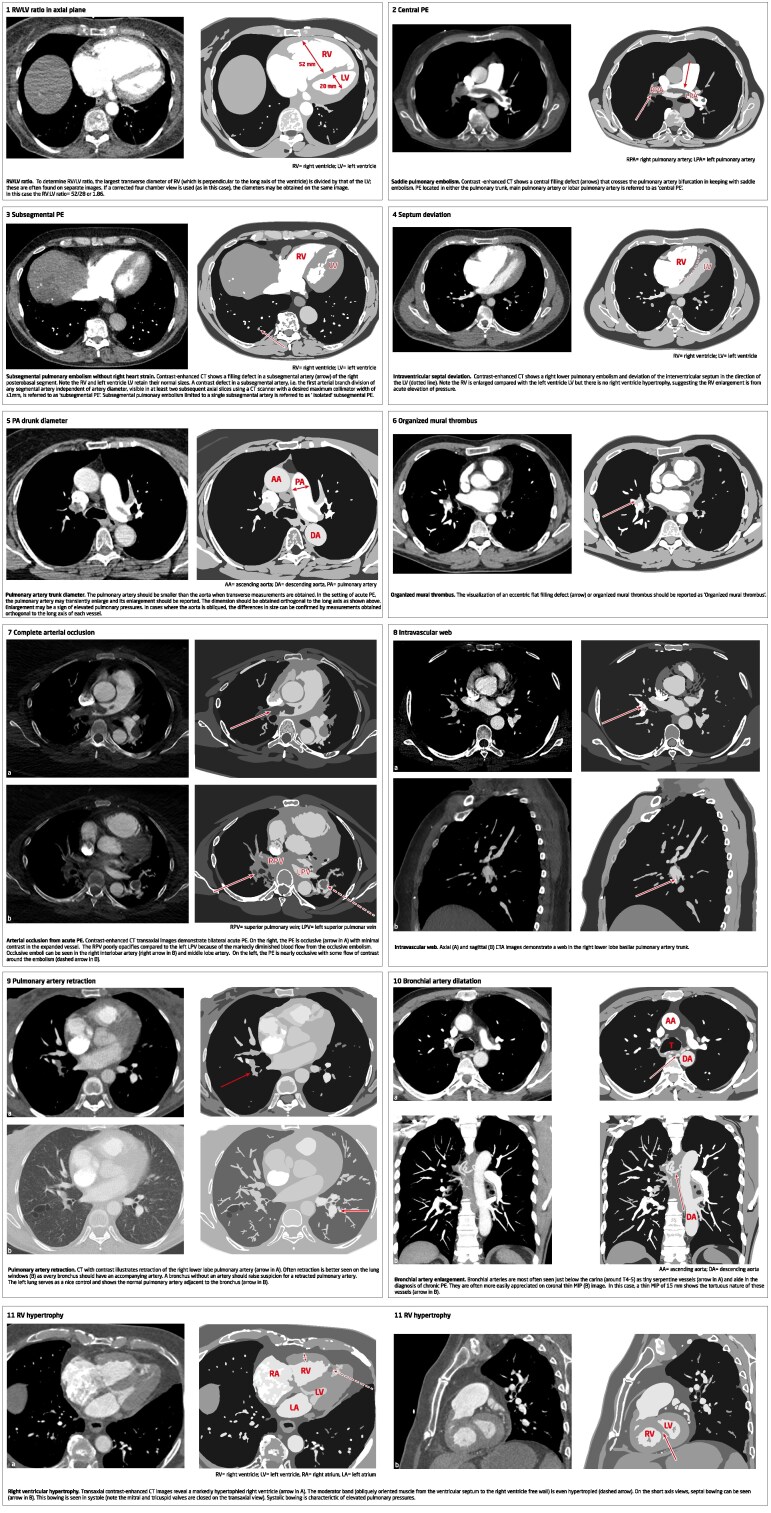
Image atlas of core set of CTA findings to be routinely mentioned in CTA reports.

**Figure 2:**
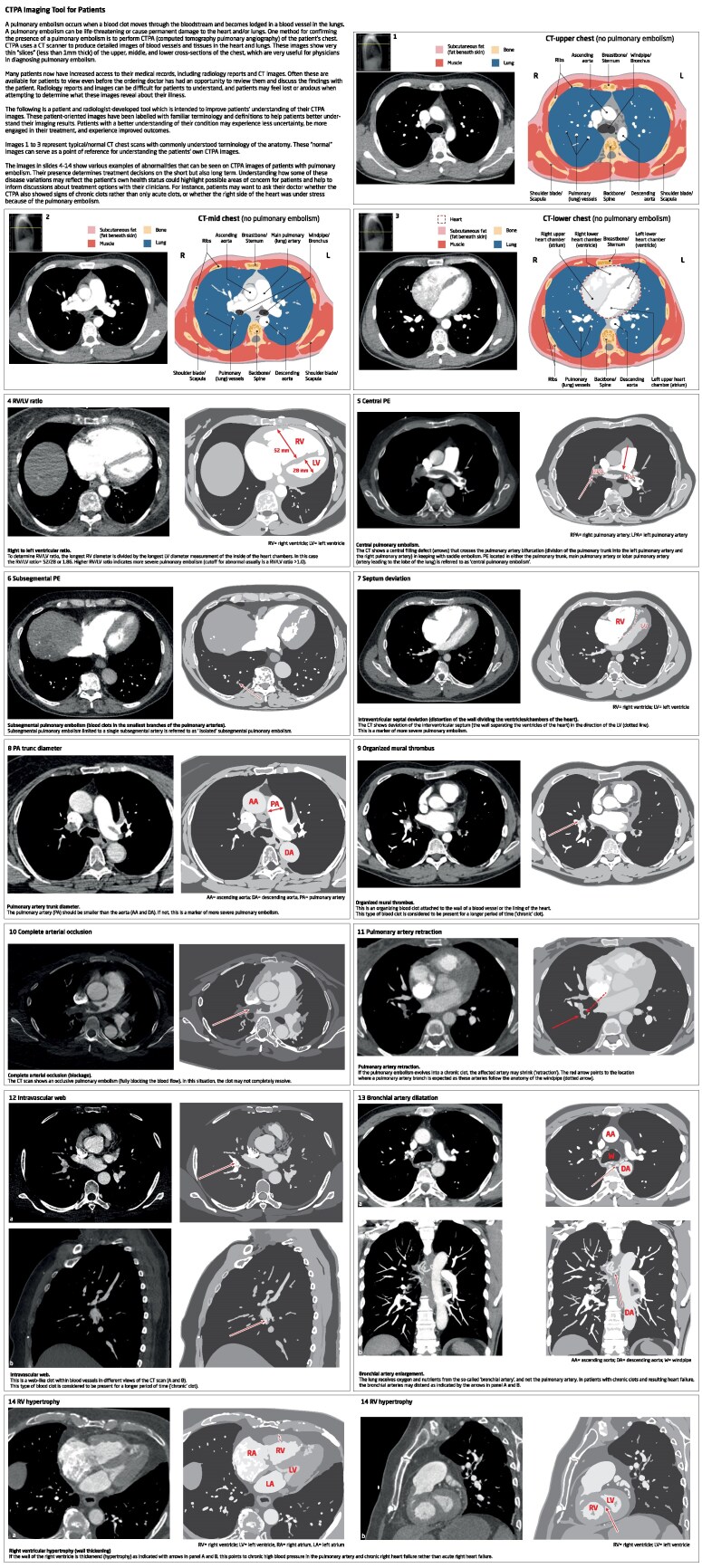
Image atlas of core set of CTA findings to be routinely mentioned in CTA reports in lay language for patients.

The selected 11 CTA findings included the following (*Figures 1 and 2*):

- the location of acute PE (central location, subsegmental PE),
- indicators of right ventricular overload which appeared to predict early haemodynamic collapse and death (RV/LV ratio, PA trunk diameter, septum deviation), and
- predictors of persistent respiratory symptoms, post-PE syndrome, and CTEPH: RV/LV ratio, PA trunk diameter, RV hypertrophy, septum deviation, organised mural thrombi, complete arterial occlusion, intravascular webs or bands, pulmonary artery retraction, and bronchial artery dilatation.

The location of the clot but mostly signs of acute RV overload are predictive of short-term adverse outcomes and death.^1,112^ Subsegmental PE may in some circumstances be left untreated.^79,113–115^ Moreover, clot location is relevant for the assessment of the suitability of the patient for percutaneous reperfusion treatment. Any additional descriptors of location or extent of clot burden may be included as ‘nice to have’ findings. These descriptors, while not useful in prognostication, may be helpful for identifying clot resolution on follow-up imaging, particularly post lytic or mechanical interventions. Of note, prognostic information of the clot cannot be estimated after resuscitation as chest compression may lead to the migration of clots in the more peripheral arteries.

The combination of several of the signs of CTEPH (more than their isolated presence at the time of index PE) pointed to the presence of a pre-existing CTEPH or its subsequent diagnosis.^70–72,77,78,116,117^ The assessment of signs of pre-existing CTEPH may allow for a more timely diagnosis of CTEPH which can be safely made after three months of effective anticoagulation. Patients with CTEPH are considered unsuitable candidates for a catheter-based mechanical embolectomy because they do not benefit, and with increasing pulmonary pressures any pulmonary vascular intervention becomes more risky.^73–76,118^

### Patient perspective

The task force included patients and caregivers from Europe and North America with personal experience with PE. They believe that understanding what it means to have a PE, and getting relevant information about our personal disease is very important. A standardized advice for reporting scans will allow all doctors to share the same, most important information among themselves and their patients, even across borders. They express the hope this new standard will give doctors and patients the best information and tools to become better partners in managing PE, improving both knowledge and outcomes.

## Conclusion

This clinical consensus statement provides a current update of CTA techniques, a definition of often used nomenclature and recommendations on the proposed content of CTA reports along with a detailed image atlas with instructions on how to assess all relevant CTA findings and a lay language guidance on the meaning of these findings. Upon implementation, this document may help to standardize CTA radiology reports across the globe and make all relevant prognostic CT findings available for treating physicians to guide management decisions, ultimately improving outcomes of care for PE patients.

## Supplementary Material

jeaf050_Supplementary_Data

## Data Availability

This is a consensus document; data availability is not applicable.
