# Supplementary material for: Optimal approach to performing and reporting computed tomography angiography for suspected acute pulmonary embolism: a clinical consensus statement of the ESC Working Group on Pulmonary Circulation & Right Ventricular Function, the Fleischner Society, the Association for Acute CardioVascular Care (ACVC) and the European Association of Cardiovascular Imaging (EACVI) of the ESC, endorsed by European Respiratory Society (ERS), Asian Society of Thoracic Radiology (ASTR), European Society of Thoracic Imaging (ESTI), and Society of Thoracic Radiology (STR)
Source: Eur Heart J Cardiovasc Imaging. 2025 Jun 3;26(7):1085–98. doi: 10.1093/ehjci/jeaf050 (PMC13376148; doi:10.1093/ehjci/jeaf050)
Supplement: jeaf050_Supplementary_Data [file jeaf050_supplementary_data.pdf]

Appendix A: search string

(("CTPA"[tw] OR "CT PA"[tw] OR "Computed Tomography Pulmonary Angiography"[tw] OR "Computed Tomography Pulmonary Angiogr*"[tw] OR "Computer Tomography Pulmonary Angiography"[tw] OR "Computer Tomography Pulmonary Angiogr*"[tw] OR "CT Pulmonary Angiography"[tw] OR "CT Pulmonary Angiogr*"[tw] OR "Computed Tomography Angiography"[Mesh] OR "Computed Tomography Angiography"[tw] OR "Computed Tomography Angiogr*"[tw] OR "Computer Tomography Angiography"[tw] OR "Computer Tomography Angiogr*"[tw] OR "CT Angiography"[tw] OR "CT Angiogr*"[tw] OR "Tomography, X-Ray Computed"[Mesh] OR "CAT Scan"[tw] OR "CAT Scans"[tw] OR "Computed Tomogr*"[tw] OR "Computed Tomography"[tw] OR "Computed X Ray Tomogr*"[tw] OR "Computed X Ray Tomography"[tw] OR "Computer Assisted Tomogr*"[tw] OR "Computer Assisted Tomography"[tw] OR "Computer Tomogr*"[tw] OR "Computer Tomography"[tw] OR "Computerised Tomogr*"[tw] OR "Computerised Tomography"[tw] OR "Computerized Tomogr*"[tw] OR "Computerized Tomography"[tw] OR "CT Scan"[tw] OR "CT Scans"[tw] OR "CT X Ray"[tw] OR "CT X Rays"[tw]) AND ("acute PE"[tw] OR "acute pulmonary embolism"[tw] OR "acute pulmonary embol*"[tw] OR "acute lung embolism"[tw] OR "acute lung embol*"[tw] OR (("Pulmonary Embolism"[mesh] OR "pulmonary embolism"[tw] OR "pulmonary embol*"[tw] OR "lung embolism"[tw] OR "lung embol*"[tw]) AND ("Acute Disease"[mesh] OR "acute"[tw]))) AND ("parameters"[tw] OR "parameter"[tw] OR "parameter*"[tw] OR "Predictive Value of Tests"[mesh] OR "predictor"[tw] OR "predictors"[tw] OR "predict"[tw] OR "Sensitivity and Specificity"[Mesh] OR "Sensitivity"[tw] OR "Specificity"[tw] OR "Reproducibility of Results"[mesh] OR "Reproducibility"[tw] OR "Reproducib*"[tw] OR "Accuracy"[tw] OR "accura*"[tw]) AND ("Outcome Assessment, Health Care"[Mesh] OR "outcome"[tw] OR "outcomes"[tw] OR "outcome*"[tw] OR "Prognosis"[Mesh] OR "Prognosis"[tw] OR "Prognostic"[tw] OR "Prognos*"[tw]) NOT (("Pulmonary Embolism/diagnosis"[Mesh] AND "diagnosis"[Subheading:NoExp]) OR "diagnosis"[ti]))

Date of search: 01-06-2022

Appendix B: Results of the Literature search focussed on short-term prognosis

Study	Characteristics of the study population	Sample size	CT parameter	Study outcome	Inter-observer variability
Norton et al. 2021[1]	CTPA-confirmed diagnosis of PE (cohort study)	245	Obstruction count	28-day all-cause death (univariate analysis: p=0.035)	Not available
			Obstruction index	28-day all-cause death (univariate analysis; p=0.042)	Not available
			Qanadli score < vs. ≥20	28-day all-cause death (difference not statistically significant)	Not available
			4-chamber RV/LV <1.0 vs. ≥1.0	28-day all-cause death (difference not statistically significant)	Not available
			IVS deviation	28-day all-cause death (difference not statistically significant)	Not available
			SVC area	28-day all-cause death (difference not statistically significant)	Not available
			PA trunk area	28-day all-cause death (difference not statistically significant)	Not available
			Azygous vein diameter	28-day all-cause death (difference not statistically significant)	Not available

			IVC reflux	28-day all-cause death (difference not statistically significant);	Not available
			Emphysema score	28-day all-cause death (univariate analysis: p=0.017)	Not available
			Coronary artery calcification score (<6 vs. ≥6)	28-day all-cause death (univariate analysis: p=0.023)<br>6-month all-cause death (multivariable analysis: aOR 2.3, 95% CI 1.1-4.8)	Not available
Becattini et al. 2021[2]	Low-risk confirmed acute PE (individual patient meta-analysis)	IPD MA<br>Short-term: 8 studies, 1479 patients<br>3-month: 9 studies, 1546 patients	RV enlargement (as defined in each individual study)	In-hospital/30-day all-cause mortality (OR 2.03, 95% CI 0.51-8.10)	Not available
Cozzi et al. 2021[3]	CTPA confirmed diagnosis of PE	780	RV/LV ratio	30-day all-cause death (HR 2.91, p=0.0007);<br>No statistical difference in multivariable analysis	Not available
			PA diameter	30-day all-cause death (HR 2.91, p=0.0092);<br>no statistical difference in multivariable analysis	Not available
			Azygos vein diameter	30-day all-cause death (HR 2.91, p=0.0385);	Not available

				no statistical difference in multivariable analysis	
			Coronary sinus diameter	30-day all-cause mortality (HR 2.91, p=0.0026); multivariable analysis (HR 2.5, 95% CI 1.1-5.6)	Not available
Chaosuwannakit 2021 et al.[4]	CTPA confirmed diagnosis of PE	238	RV diameter	30-day mortality: aOR 1.094 (95% CI 1.007-1.188)	ICC 0.997, 95% CI 0.91-0.998
			CT obstruction index (Qanadli)	30-day mortality: aOR 1.040 (95% CI 1.003-1.079)	ICC 0.984, 95% CI 0.91-0.991
Van Dam 2021 et al.[5]	Hemodynamically stable adult patients with CTPA-confirmed acute symptomatic PE	100	RV/LV ratio	ICU admission at 7 days: aOR 6.27 (95% CI 0.88-44.6)<br>Reperfusion therapy at 7 days: aOR 65.4 (95% CI 0.24-1760)<br>PE-related mortality at 7 days: N/A	Not available
			PA trunk diameter	ICU admission at 7 days: aOR 1.03 (95% CI 0.83-1.28)<br>Reperfusion therapy at 7 days: aOR 1.51 (95% CI 0.87-2.63)<br>PE-related mortality at 7 days: N/A	Not available

			Total thrombus obstruction score (Qanadli)	ICU admission at 7 days: aOR 1.07 (95% CI 0.99-1.15)<br>Reperfusion therapy at 7 days: aOR 1.21 (95% CI 0.98-1.49)<br>PE-related mortality at 7 days: N/A	Not available
			Perfusion defect score (CTPP)	ICU admission at 7 days: aOR 0.96 (95% CI 0.86-1.06)<br>Reperfusion therapy at 7 days: aOR 1.19 (95% CI 0.95-1.49)<br>PE-related mortality at 7 days: N/A	Not available
Jia et al. 2019[6]	Non-high risk with CTPA confirmed acute PE	341	Decision tree including RV/LV ratio, central pulmonary artery embolism, RA/LA ratio	Predictive ability for the composite endpoint of death; cardiopulmonary resuscitation; endotracheal intubation; vasopressor requirement for systemic hypotension (more than 5 µg per kilogram); or reperfusion treatment to save the patient's life: AUC 0.858 (95% CI 0.775-0.941)	Not available

			Decision tree including the obstruction area index	Predictive of death; cardiopulmonary resuscitation; endotracheal intubation; vasopressor requirement for systemic hypotension (more than 5 µg per kilogram); or reperfusion treatment to save the patient's life: AUC 0.810 (95% CI 0.706-0.913)	Not available
Osman et al. 2018[7]	Acute PE	150	PA obstruction index (Qanadli)	30-day all-cause mortality (45% cut-off): sensitivity 60%, specificity 80%	Not available
Alonso Martinez et al. 2016[8]	Hemodynamically stable adult patients with CTPA-confirmed acute symptomatic PE	530	PA obstruction index (Qanadli)	The thrombi burden of dead patients was 33.75% (IQR: 25) while the thrombi burden of survivors was 30% (IQR: 32.25) (P < .001).	Not available
			Central thrombi (main trunk and/or RPA or LPA)	Death: aOR 1.31 (95% CI 1.007-3)	Not available
Aviram et al. 2016[9]	CTPA confirmed acute PE	756	LV volume	30-day death: 0.987 (0.978-0.997)	Not available
			RV volume	30-day death: 0.995 (0.989-1.002)	Not available

			LA volume	30-day death: 0.985 (0.975-0.994)	Not available
			RA volume	30-day death: 0.995 (0.989-1.002)	Not available
			RV/LV volume ratio	30-day death: 1.119 (0.904-1.384)	Not available
			RA/LA volume ratio	30-day death: 1.181 (0.894-1.561)	Not available
			LA volume (cut-off 62 ml)	30-day death: aHR 2.44 (95% CI 1.52-3.9)	Not available
			RA/LA volume ratio (cut-off 1.2)	30-day death: aHR 2.1 (95% CI 1.32-3.37)	Not available
			LV volume (cut-off 67 ml)	30-day death: aHR 1.8 (95% CI 1.08-3.01)	Not available
Meyer et al. 2022[9]	CTPA confirmed acute PE	234	Low skeletal muscle mass (skeletal muscle index)	30-day death (multivariate): HR 1.08, 95% CI 1.04-1.11)	Not available
Bach et al. 2015[10]	CTPA confirmed acute PE	365	IVC reflux	30-day deathaOR 3.29, p=0.001	Not available
			PA diameter	Significantly greater values in non-survivors, p=0.016	Not available
			RV/LV ratio	Not significantly greater values in non-survivors, p=0.078	Not available
George et al. 2014[11]	CTPA confirmed acute PE with echo exam	804	RV/LV ratio	30-day deathaOR 1.14, 95% CI 1.02-1.27	Not available

Vedovati et al. 2013[12]	CTPA confirmed acute PE	Study-level meta-analysis, 16 studies, 3884 patients	Obstruction index (Qanadli)	30-day deathOR 1.78, 95% CI 1.08-2.93	Not available
		Study-level meta-analysis, 5 studies, 2215 patients	Central localization of emboli	30-day death: OR 2.51, 95% CI 0.88-7.15	Not available
Jimenez et al. 2014[13]	Hemodynamically stable adult patients with CTPA-confirmed acute symptomatic PE	848	RV/LV ratio >0.9	30-day death incidence: absolute difference 0.4%, 95% CI -2.5% - +3.3%, p=0.932; 30-day PE-related death incidence: absolute difference 1.6%, 95% CI +0.3% - +2.9%, p=0.053 Hemodynamic collapse: absolute difference 1.6%, 95% CI -0.8% - +4.0%, p=0.293	Not available
Venkatesh et al. 2010[14]	CTPA-confirmed acute symptomatic PE	125	Obstruction score (Qanadli)	30-day PE-related death: OR 0.85, 95% CI 0.75-0.96 Adjusted OR 1.1, 95% CI 0.94-1.25	Not available
			Obstruction score (Mastora)	30-day PE-related death: OR 0.95, 95% CI 0.92-0.98 Adjusted OR 0.95, 95% CI 0.9-1.01	Not available

			Obstruction score (Central)	30-day PE-related death: OR 0.92, 95% CI 0.87-0.97<br>Adjusted OR 1.01, 95% CI 1.03-1.16	Not available
			RV diameter (maximum)	30-day PE-related death: OR 0.95, 95% CI 0.88-1.02	Not available
			LV diameter (maximum)	30-day PE-related death: OR 1.09, 95% CI 1.01-1.18	Not available
			RV/LV	30-day PE-related death: OR 0.41, 95% CI 0.15-1.1	Not available
			IVS deviation	30-day PE-related death: OR 0.85, 95% CI 0.75-0.96	Not available
Schoepf et al. 2004[15]	CTPA confirmed acute PE	431	RV/LV ratio >0.9	30-day death: Adj HR 5.2 (1.6-16.4)<br>30-day death and in-hospital ccomplications (CPR, MV, vasopressors, lytics, catheter intervention or surgical embolectomy): Adj HR 2.4 (1.5-3.8)	Not available
van der Meer et al. 2005[16]	CTPA confirmed acute PE		RV/LV ratio $\leq$ 1.0 / 1.0-1.5 / >1.5	90-day PE related death<br>Higher RV/LV ratio in PE deaths (1.5+/-0.2 vs. 1.1+/- 0.03), p 0.005.	Not available

				Risk of death 10% if ratio > 1.0 vs. 0% if <1.0, no stats	
			PA to Ao diameter	No relationship between ratio and mortality (p 0.66)	Not available
			Obstruction index (Qanadli)	Sigificantly higher obstruction index in 90-day PE related death (60+/-28) vs. survivors (29+/-21), p<.001 or non PE deaths (37+/-26), p.027<br>HR for index >40%11.2 (1.3-93.6)	Not available
			Septum displacement (flattening or converse toward LV)	No association with h90-day PE related death (p 0.20)	Not available
Rotzinger et al. 2020[17]	CTPA proven non massive acute PE	690	Obstruction index (Qanadli)	30-day mortality: no significant association with OI>40%: OR 1.3 (0.7-2.6) or with OI<20%: OR 0.5 (0.2-1.3)<br>90-day mortality: significant increase in OI>40%: OR 2.0 (1.1-3.6), and with OI<20%: OR 0.6 (O.3-0.9)	Not available

				Subgroup analyses showing that results were driven by patients without cardiopulm disease. No assoc. in those with CPD.	
Kwak et al. 2013[18]	Non-high risk CTPA confirmed PE	297	Saddle PE	30-day mortality: no significant association (18.5% vs 11.9%, p 0.32) Thrombolytic more often administered (29.6% vs 8.1%, p<0.001) 30-day Major adverse events (PE related shock, MV, in hospital mortality, thrombolysis and thrombectomy)59.3 vs25.6%, p<0.01	Not available
Lyhne et al. 2019[19]	CTPA confirmed PE	302	RV/LV ratio >1.0	5-day deterioriation (all cause mortality, need for rescue intervn, unstable dysrhythmia, need for hemodynamic support, need for respiratory support): OR 1.3 (0.6-3.0)	Pearson absolute value 0.81
			Septal bowing	OR 2.2 (1.1-4.5), p 0.03 with single rater	Kappa 0.45

				No assoc with both raters (results not shown)	
			Contrast in IVC	No assoc (results not shown)	Kappa 0.83
			Main/Rt/Lt PA diameter	OR 1.08 (1.01-1.16), p=0.027<br>Rt and Lt NS	Pearson IOR: 0.86/0.86/0.90
Beenen et al. 2018[20]	CTPA confirmed PE	1950	RV/LV >1.0	Mortality at 1 week / 1 monthh / 1 year: NS	Announced but results not provided
			RV/LV short axis > 0.9	NS	
			PA/Ao ratio >1.0	NS	
			PA diameter > 29 mm	4.2 (1.0-16.8) at one week<br>2.3 (1.4-4.0) at one year	
			Cardiothoracic ratio > 0.5	NS	
			Septal bowing (neg/neutral/pos)	NS	
			Contrast in IVC (no, IVC only, intrahep v < 3 cm, intrahep v > 3 cm)	NS	
			Contrast in azygos	NS	
Etesamifard et al. 2016[21]	CTPA confirmed normotensive PE	190		30-day PE related death: all NS<br>30-day composite lf death, hemodynamic instability, thrombolysis,	

				thrombectomy, inotrop, mechanical ventilation. Longterm all-cause mortality	
			RV/LV ratio	30-day composite OR 2.8 (1.5-5.3), 3.0 (1.5-6.3) in multiv	
			SVC diameter	30-day composite 1.1 (1.0-1.3)	
			Azygos diameter	NS	
			Contrast reflux in IVC at the leverl of the liver	30-day composite OR 7.0 (1.9-25.2)	
			Contrast reflux in azygos	NS	
			Central clot score	NS	
			Saddle/central/peripheral PE	NS	
Kang et al. 2011[22]	Haemodynamically stable hospitalized patients with CTPA confirmed PE	260		Adverse clinical outcome, defined as: death within 30 days or escalation of therapy according to the MAPPET-3 criteria	
			Septal bowing (normal vs flattend-spetal bowing)	Adverse outcome HR: 2.07 (95%CI 1.22-3.52, p=0.007)<br>30d death HR: NS	Kappa= 0.44

			IVC reflux (none- trace IVC only- IVC but not hepatic veins- IVC proximal hepatic veins – IVC hepatic veins mid portion of the liver- IVC opacification distal hepatic veins)	Adverse outcome HR: 2.57 (95%CI 1.47-4.50, p=0.001)<br>30d death HR: NS	Kappa=0.68
			RV/LV ratio (diameter axial view)	AUC for AE= 0.658 (95% CI: 0.597 to 0.715)<br>AUC for 30d death= 0.698 (95% CI: 0.638 to 0.753)<br><br>Cut-off (30d death) >1.0<br>Adverse outcome HR: NS<br>30d death HR: NS	Kappa= 0.88
			RV/LV ratio (diameter 4 chamber view)	AUC for AE= 0.659 (95% CI: 0.598 to 0.717)<br>AUC for 30d death= 0.694 (95% CI: 0.634 to 0.750)<br><br>Cut-off (30d death)>1.0<br>Adverse outcome HR: 2.51 (95%CI 1.26-4.99, p=0.009)<br>30d death HR: 3.68 (95% CI: 1.08 to 12.60, p=0.039)	Kappa=0.85

			RV/LV ratio (3D volume)	AUC for AE= 0.677 (95% CI: 0.617 to 0.734)<br>AUC for 30d death= 0.664 (95% CI: 0.603 to 0.721)<br><br>Cut-off (30d death) >1.2<br><br>Adverse outcome HR: 4.04 (95%CI 2.0-8.16, p<0.001)<br>30d death HR: 6.49 (95% CI: 1.77 to 23.84, p=0.005)	Kappa= 0.93
Araoz et al. 2003[23]	CTPA confirmed PE	173		Univariate analysis of death by PE, death by any cause, cardiac arrest, intubation, vasopressors, ICU admission<br><br>Multivariate analysis corrected for congestive heart failure, ischemic heart disease and pulmonary disease	Not available

			Ventricular septal bowing (straightened or bowed)	Univariate all NS except for ICU admission (p=0.004)<br>Multivariate for ICU admission OR 2.8 (95%CI 1.3-6.03, p=0.009)<br>Multivariate for vasopressors OR 1.08 (95%CI 1.01-1.14, p=0.015)	Not available
			RV-LV ratio	Univariate all NS except for ICU admission (p=0.025)<br>Multivariate for ICU admission OR 3.61 (95%CI 1.64-7.98, p=0.003)	Not available
			Clot burdon (minor variation to Qanadli score)	Univariate all NS exept for vasopressors (p=0.046)	Not available
			PA/AO diameter	Univariate all NS	Not available
			Oligemia right lung	Univariate all NS exept for intubation (p=0.0009) and vasopressor (p=0.020)	Not available
			Oligemia left lung	Univariate all NS exept for intubation (p=0.0089)	Not available

Karri et al 2020[24]	CTPA confirmed PE	102		Hemodynamic failure, ICU admission, vasopressor requirement, mechanical ventilation, thrombolytic use, attributable death to PE	Not available
			PA trunk > 3cm	Univariate analysis all NS Multivariate analysis all NS	K=0.89
			Paradoxical septal bowing	Univariate analysis all NS Multivariate analysis all NS exept for thrombolytic use (p=0.02)	K=0.76
			RV/LV ratio > 1	Univariate analysis all NS Multivariate analysis all NS	K=0.76
			IVC contrast reflux	Univariate analysis all NS Multivariate analysis all NS exept for PE related death (p=0.03)	K=0.75
			Hepatic vein contrast reflux	Univariate analysis all NS Multivariate analysis all NS	K=0.92
Ende-Verhaar et al. 2017[25]	Normotensive CTPA confirmed PE		RV/LV ratio >1	NA	Cohen Kappa between internal medicine residents and a experience

					radiologist: 0.86 (95%CI 0.75–0.96), 0.94 (95%CI 0.87–1.00) and 0.83 (95%CI 0.72–0.94)<br><br>Cohen Kappa statistics between the residents internal medicine were 0.88 (95%CI 0.78–0.97;), 0.85 (95%CI 0.75–0.96;) and 0.85 (95%CI 0.75–0.96;)
Kumamaru et al. 2016[26]	CTPA confirmed PE	1698		30 d all cause mortality and 30d PE-related mortality	
			Most proximal ambolus	NS	Inter-reader agreement 95.2%
			Latereralty of PE	NS	Inter-reader agreement 98.9%
			Parenchymal infarction	NS	Inter-reader agreement 98.9%
			RV-LV ratio >1	34.5% no 30-d PE related death vs 50.0% PE related death (p=0.024) OR: 1.89	Inter-reader agreement 95.2%

			Contrast reflux to the hepatic portion of IVC	21.7% no 30-d PE related death vs 35.1 PE related death (p=0.006)<br>OR (self-calc): 1.96	Inter-reader agreement 97.4%
			Bowing of the septum	NS	Inter-reader agreement 92.3%
Kumamaru et al. 2012[27]	CTPA confirmed PE	200	4ch-1 was a single oblique technique using<br>LV morphology landmarks	AUC 30d PE related death: 0.55<br>AUC 30d PE related death or intensive therapie: 0.65	r= 0.902
			4ch-2 oblique techniquesthat created an intermediate shortaxis image to identify the maximum RV diameter but with different approaches to reach short-axis images	AUC 30d PE related death 0.69<br>AUC 30d PE related death or intensive therapie: 0.77<br>Significant higher ratio in cases with 30d PE related death or intensive therapies (p<0.001)	r=0.920
			4ch-3 oblique technique that created an intermediate shortaxis image to identify the maximum RV diameter but with different approaches to reach short-axis images	AUC 30d PE related death 0.70<br>AUC 30d PE related death or intensive therapie: 0.78<br>Significant higher ratio in cases with 30d PE related death or	r=0.925

				intensive therapies (p<0.001)	
			Axiaal	AUC 30d PE related death 0.73<br>AUC 30d PE related death or intensive therapie: 0.78<br>Significant higher ratio in cases with 30d PE related death or intensive therapies (p<0.001)	r=0.881
Araoz et al. 2007[28]	CTPA confirmed PE	1056	Ventricular septal bowing	death due to PE for observer 1 (OR, 1.98;P=.04) and for observer 2 (OR, 1.52;P=0.22)<br>Multivariate analysis for observer 1: OR 1.97 (p=0.05) or observer 2: OR 1.59 (p=0.19)	K= 0.54
			RV-LV ratio	death due to PE for observer 1 (OR, 1.00;P=.99) and for observer 2 (OR, 1.20;P=0.52)	K=0.51
			Embolic burden (minor adjusted from Qanadli score)	death due to PE for observer 1 (OR, 0.98;P=.31) and for	K=0.85

				observer 2 (OR, 0.95;P=0.02)	
Pech et al. 2007[29]	CTPA confirmed PE	694	Clot burdon (PE index= sum of Nseg x D)	Logistic regression models demonstrated that the PE index was not a significant predictor of patient death.	Kendall-W-test used for calculation of interobserver agreement showed a correlation rate for the assessment of PE index of 0.993 (p<0.001)

Appendix C: Results of the Literature search focussed on long-term prognosis

Study	Characteristic of the study population	Sample size	CT parameter	Time of follow-up	Study outcome	Inter-observer variability
Norton et al. 2021[1]	CTPA-confirmed diagnosis of PE (cohort study)	245	Obstruction count	6 months	Statistical difference in 6-month all-cause death rate (univariate analysis)	Not available
			Obstruction index	6 months	Statistical difference in 6-month all-cause death rate (univariate analysis)	Not available
			Qanadli score < vs. ≥20	6 months	6-month all-cause death (difference not statistically significant)	Not available
			4 chamber RV:LV (< vs. ≥1.0 ratio)	6 months	6-month all-cause death (difference not statistically significant)	Not available
			IVS deviation	6 months	6-month all-cause death (difference not statistically significant)	Not available
			SVC area	6 months	6-month all-cause death (difference not statistically significant)	Not available
			PA trunk area	6 months	6-month all-cause death (difference not statistically significant)	Not available
			Azygous vein diameter	6 months	6-month all-cause death (difference not statistically significant)	Not available

			IVC reflux	6 months	6-month all-cause death (difference not statistically significant)	Not available
			Emphysema score	6 months	Statistical difference in 6-month all-cause death rate (univariate analysis)	Not available
			Coronary artery calcification score (<6 vs. ≥6)	6 months	Statistical difference in 6-month all-cause death rate (univariate and multivariable analysis)	Not available
Becattini et al. 2021[2]	Low-risk confirmed acute PE (individual patient meta-analysis)	8 studies, 1479 patients 3-month: 9 studies, 1546 patients	RV enlargement by CT (defined in individual studies)	3 months	3-month all-cause death (OR 2.37, 95% CI 0.77-7.31)	Not available
Aranda et al. 2021[30]	Acute PE	6-month FUP (cohort study)	Presence of COPD	6 months	53.6% vs. 77% thrombus resolution: OR 2.9 (95% CI 1.29–6.51)	Not available
			Active cancer	6 months	88.9% vs. 70.9% thrombus resolution: OR 3.28 (95% CI 1.23–8.74)	Not available

			Unprovoked PE	6 months	37.1% vs. 55.9% thrombus resolution; OR 2.15 (95% CI 1.19–3.89)	Not available
			RV/LV >1	6 months	54.8% vs. 78.2% thrombus resolution; OR 0.34 (95% CI 0.17–0.68)	Not available
			Treatment <7 days	6 months	78% vs. 65.3% thrombus resolution; OR 1.89 (95% CI 1.03–3.46)	Not available
			Qanadli score	6 months	12.3 vs. 18.3 thrombus resolution; OR 2.02 (95% CI 1.35–9.32)	Not available
			Qanadli score ≥16	6 months	64.3% vs. 78.2% thrombus resolution; OR 0.50 (95% CI 0.27–0.92)	Not available
			Residual pulmonary obstruction	6 months	In participants without indefinite or prolonged anticoagulation treatment, OR for the association with recurrent PE 4.67 (95% CI, 1.26–17.26)	Not available
			Right ventricular overload (RV/LV diameter ratio above 1)	6 months	OR 10.3 (95% CI 1.8– 58) for CTEPH	Not available

Vedovati et al. 2013[12]	CTPA-confirmed acute PE	Study-level meta-analysis (16 studies and 3884 patients)	Obstruction index (Qanadli)	3 months	3-month death: OR 1.86, 95% CI 1.27-2.71	Not available
Beenen et al. 2018[20]	Acute PE	n=1950 (post-hoc analysis of trials)	RV/LV >1; RV/LVsa >0.9; Septal bowing; Septal flattening; Aorta >40 mm; Pulmonary trunk >29 mm; PT/Ao >1.0; Cardiothoracic ratio >0.50; Backflow IVC; Intrahepatic reflux; Backflow azygos vein	1 year	1-year death: association only with Pulmonary trunk >29 mm	Not available
			RV/LV >1; RV/LVsa >0.9;	1 year	1-year adverse events: no association	Not available

			Septal bowing; Septal flattening; Aorta >40 mm; Pulmonary trunk >29 mm; PT/Ao >1.0; Cardiothoracic ratio >0.50; Backflow IVC; Intrahepatic reflux; Backflow azygos vein			
Mean et al 2017[31]	Acute PE	291 patients aged >65 years, Cohort study	CTOI (per 10%)	90 days	OR 0.92 (0.70; 1.21) for 90-day death	Not available
			RV/LV diameter ratio (per unit)	90 days	OR 0.35 (0.06; 2.18) for 90-day death	
			CTOI (per 10%)	Long-term FUP	OR 1.36 (1.03; 1.81) for PE-related death	
			RV/LV diameter ratio (per unit)	Long-term FUP	OR 0.69 (0.23; 2.07) for PE-related death	

			CTOI (per 10%)	Long-term FUP	OR 1.27 (1.12; 1.45) for VTE recurrence	
			RV/LV diameter ratio (per unit)	Long-term FUP	OR 2.74 (1.26; 5.95) for VTE recurrence	
Alonso Martinez et al 2016[8]	Acute PE	530 (cohort study)	Central location of thrombi	Long-term FUP	OR 1.31 (CI 95% 1.007-3) for death (any time)	
Etesamifard et al, 2016[21]	Normotensive PE	190 (cohort study)	RV/LV ratio	Long-term FUP	HR: 1.762, 95 % CI 0.968-4.218; p value = 0.064 for long-term mortality	
Rotzinger et al 2020[17]	Acute PE	705 (cohort study)	Qanadli CT obstruction index	Long-term FUP	In patients with cardiopulmonary disease, CTOI associated with higher mortality (if CTOI>40%) and lower mortality (if CTOI<20)	
Den Exter et al 2015[32]	Acute PE	157 (cohort study)	Qanadli CT obstruction index	Long term	Residual thromboembolic obstruction not associated with recurrent VTE (aHR: 0.92; 95 % CI: 0.2-4.1)	
Van Dam et al 2021[5]	Acute PE	97 (cohort study)	Perfusion defect score (PDS)	Long-term	PDS not correlated to persistent dyspnea (mean difference −3.7%; 95%CI −9.7% to 2.3%), chest pain (mean difference −0.70%; 95%CI −8.7% to 7.3%) or post-PE	

					functional impairment (mean difference −4.7%; 95%CI −11% to 1.3%,	
van der Meer et al. 2005[16]	CTPA confirmed acute PE		RV/LV ratio $\leq$ 1.0 / 1.0-1.5 / >1.5	90 days	90-day PE related death<br>Higher RV/LV ratio in PE deaths (1.5+/-0.2 vs. 1.1+/- 0.03), p 0.005.<br>Risk of death 10% if ratio > 1.0 vs. 0% if <1.0, no stats	Not available
			PA to Ao diameter	90 days	No relationship between ratio and risk of death (p=0.66)	Not available
			Obstruction index (Qanadli)	90 days	Significantly higher obstruction index in 90-day PE-related death (60+/-28) vs. survivors (29+/-21), p<.001 or non PE deaths (37+/-26), p.027<br>HR for index >40%11.2 (1.3-93.6)	Not available
Ende Verhaar et al, 2019[33]	CTPA-confirmed acute PE	100 patients	intravascular webs; pulmonary artery retraction or dilatation; bronchial artery dilatation; right	>3 months	Multivariable analysis identified 6 radiologic parameters as independent predictors of CTEPH: The presence of 3 or more these parameters was associated with a sensitivity of 70% (95% CI 55%-	

			ventricular hypertrophy; and interventricular septum flattening.		82%), a specificity of 96% (95% CI 86%-100%), and a c-statistic of 0.92.	
Barco et al; 2022[34]	CTPA-confirmed acute PE (cohort study)	303 patients	Intravascular webs, arterial narrowing or retraction, dilated bronchial arteries, and right ventricular hypertrophy	2 years	CTEPH in 11.0% of the patients with at least two signs of chronic pulmonary PE plus one sign of chronic pulmonary hypertension (expert 1), or in 4.0% of the patients exhibiting the same number of signs (expert 2);<br>CTEPH in 9.8% per expert 1 and 4.5% per expert in the presence of three of six signs according to[33]	K: intravascular webs 0.313; complete arterial occlusion 0.249; arterial narrowing or retraction 0.316; dilated bronchial arteries 0.348; right ventricular hypertrophy 0.223.

References

1. Norton L, Cooper G, Sheerins O, et al. Clinical and radiological characteristics of acute pulmonary embolus in relation to 28-day and 6-month mortality. *PloS one* 2021; **16**(12): e0258843.

2. Becattini C, Maraziti G, Vinson DR, et al. Right ventricle assessment in patients with pulmonary embolism at low risk for death based on clinical models: an individual patient data meta-analysis. *European heart journal* 2021; **42**(33): 3190-9.

3. Cozzi D, Moroni C, Cavigli E, et al. Prognostic value of CT pulmonary angiography parameters in acute pulmonary embolism. *Radiol Med* 2021; **126**(8): 1030-6.

4. Chaosuwannakit N, Soontrapa W, Makarawate P, Sawanyawisuth K. Importance of computed tomography pulmonary angiography for predict 30-day mortality in acute pulmonary embolism patients. *Eur J Radiol Open* 2021; **8**: 100340.

5. van Dam LF, Kroft LJM, Boon G, Huisman MV, Ninaber MK, Klok FA. Computed tomography pulmonary perfusion imaging and 3-months clinical outcomes after acute pulmonary embolism. *Thrombosis research* 2021; **199**: 32-4.

6. Jia D, Li XL, Zhang Q, Hou G, Zhou XM, Kang J. A decision tree built with parameters obtained by computed tomographic pulmonary angiography is useful for predicting adverse outcomes in non-high-risk acute pulmonary embolism patients. *Respir Res* 2019; **20**(1): 187.

7. Osman AM, Abdeldayem EH. Value of CT pulmonary angiography to predict short-term outcome in patient with pulmonary embolism. *Int J Cardiovasc Imaging* 2018; **34**(6): 975-83.

8. Alonso Martinez JL, Anniccherico Sánchez FJ, Urbieta Echezarreta MA, García IV, Álvaro JR. Central Versus Peripheral Pulmonary Embolism: Analysis of the Impact on the Physiological Parameters and Long-term Survival. *N Am J Med Sci* 2016; **8**(3): 134-42.

9. Aviram G, Soikher E, Bendet A, et al. Prediction of Mortality in Pulmonary Embolism Based on Left Atrial Volume Measured on CT Pulmonary Angiography. *Chest* 2016; **149**(3): 667-75.

10. Bach AG, Nansalmaa B, Kranz J, et al. CT pulmonary angiography findings that predict 30-day mortality in patients with acute pulmonary embolism. *European journal of radiology* 2015; **84**(2): 332-7.

11. George E, Kumamaru KK, Ghosh N, et al. Computed tomography and echocardiography in patients with acute pulmonary embolism: part 2: prognostic value. *Journal of thoracic imaging* 2014; **29**(1): W7-12.

12. Vedovati MC, Germini F, Agnelli G, Becattini C. Prognostic role of embolic burden assessed at computed tomography angiography in patients with acute pulmonary embolism: systematic review and meta-analysis. *Journal of thrombosis and haemostasis : JTH* 2013; **11**(12): 2092-102.

13. Jiménez D, Lobo JL, Monreal M, et al. Prognostic significance of multidetector CT in normotensive patients with pulmonary embolism: results of the protect study. *Thorax* 2014; **69**(2): 109-15.

14. Venkatesh SK, Wang SC. Central clot score at computed tomography as a predictor of 30-day mortality after acute pulmonary embolism. *Ann Acad Med Singap* 2010; **39**(6): 442-7.

15. Schoepf UJ, Kucher N, Kipfmueller F, Quiroz R, Costello P, Goldhaber SZ. Right ventricular enlargement on chest computed tomography: a predictor of early death in acute pulmonary embolism. *Circulation* 2004; **110**(20): 3276-80.

16. van der Meer RW, Pattynama PM, van Strijen MJ, et al. Right ventricular dysfunction and pulmonary obstruction index at helical CT: prediction of clinical outcome during 3-month follow-up in patients with acute pulmonary embolism. *Radiology* 2005; **235**(3): 798-803.

17. Rotzinger DC, Knebel JF, Jouannic AM, Adler G, Qanadli SD. CT Pulmonary Angiography for Risk Stratification of Patients with Nonmassive Acute Pulmonary Embolism. *Radiol Cardiothorac Imaging* 2020; **2**(4): e190188.

18. Kwak MK, Kim WY, Lee CW, et al. The impact of saddle embolism on the major adverse event rate of patients with non-high-risk pulmonary embolism. *Br J Radiol* 2013; **86**(1032): 20130273.

19. Lyhne MD, Schultz JG, MacMahon PJ, et al. Septal bowing and pulmonary artery diameter on computed tomography pulmonary angiography are associated with short-term outcomes in patients with acute pulmonary embolism. *Emerg Radiol* 2019; **26**(6): 623-30.

20. Beenen LFM, Bossuyt PMM, Stoker J, Middeldorp S. Prognostic value of cardiovascular parameters in computed tomography pulmonary angiography in patients with acute pulmonary embolism. *The European respiratory journal* 2018; **52**(1).

21. Etesamifard N, Shirani S, Jenab Y, Lotfi-Tokaldany M, Pourjafari M, Jalali A. Role of clinical and pulmonary computed tomography angiographic parameters in the prediction of short- and long-term mortality in patients with pulmonary embolism. *Internal and emergency medicine* 2016; **11**(3): 405-13.

22. Kang DK, Thilo C, Schoepf UJ, et al. CT signs of right ventricular dysfunction: prognostic role in acute pulmonary embolism. *JACC Cardiovascular imaging* 2011; **4**(8): 841-9.

23. Araoz PA, Gotway MB, Trowbridge RL, et al. Helical CT pulmonary angiography predictors of in-hospital morbidity and mortality in patients with acute pulmonary embolism. *Journal of thoracic imaging* 2003; **18**(4): 207-16.

24. Karri J, Truong T, Hasapes J, et al. Correlating computed tomography pulmonary angiography signs of right ventricular strain in pulmonary embolisms to clinical outcomes. *Ann Thorac Med* 2020; **15**(2): 64-9.

25. Ende-Verhaar YM, Kroft LJM, Mos ICM, Huisman MV, Klok FA. Accuracy and reproducibility of CT right-to-left ventricular diameter measurement in patients with acute pulmonary embolism. *PloS one* 2017; **12**(11): e0188862.

26. Kumamaru KK, Saboo SS, Aghayev A, et al. CT pulmonary angiography-based scoring system to predict the prognosis of acute pulmonary embolism. *J Cardiovasc Comput Tomogr* 2016; **10**(6): 473-9.

27. Kumamaru KK, Hunsaker AR, Wake N, et al. The variability in prognostic values of right ventricular-to-left ventricular diameter ratios derived from different measurement methods on

computed tomography pulmonary angiography: a patient outcome study. *Journal of thoracic imaging* 2012; **27**(5): 331-6.

28. Araoz PA, Gotway MB, Harrington JR, Harmsen WS, Mandrekar JN. Pulmonary embolism: prognostic CT findings. *Radiology* 2007; **242**(3): 889-97.

29. Pech M, Wieners G, Dul P, et al. Computed tomography pulmonary embolism index for the assessment of survival in patients with pulmonary embolism. *European radiology* 2007; **17**(8): 1954-9.

30. Aranda C, Gonzalez P, Gagliardi L, Peralta L, Jimenez A. Prognostic factors of clot resolution on follow-up computed tomography angiography and recurrence after a first acute pulmonary embolism. *Clin Respir J* 2021; **15**(9): 949-55.

31. Méan M, Tritschler T, Limacher A, et al. Association between computed tomography obstruction index and mortality in elderly patients with acute pulmonary embolism: A prospective validation study. *PloS one* 2017; **12**(6): e0179224.

32. den Exter PL, van Es J, Kroft LJ, et al. Thromboembolic resolution assessed by CT pulmonary angiography after treatment for acute pulmonary embolism. *Thrombosis and haemostasis* 2015; **114**(1): 26-34.

33. Ende-Verhaar YM, Meijboom LJ, Kroft LJM, et al. Usefulness of standard computed tomography pulmonary angiography performed for acute pulmonary embolism for identification of chronic thromboembolic pulmonary hypertension: results of the InShape III study. *J Heart Lung Transplant* 2019; **38**(7): 731-8.

34. Barco S, Mavromanoli AC, Kreitner KF, et al. Preexisting Chronic Thromboembolic Pulmonary Hypertension in Acute Pulmonary Embolism. *Chest* 2023; **163**(4): 923-32.
